# Non-Technical Skills in Social Networks: The Spread of Safety Communication and Teamwork in a Warehouse

**DOI:** 10.3390/ijerph18020467

**Published:** 2021-01-08

**Authors:** Alessio Paolucci, Sergio Sangiorgi, Marco Giovanni Mariani

**Affiliations:** 1 Department of Education Studies “Giovanni Maria Bertin”, Alma Mater Studiorum—University of Bologna, 40126 Bologna, Italy; alessio.paolucci2@unibo.it; 2 Unveil Consulting, 48018 Faenza, Italy; sergio.sangiorgi@unveilconsulting.com; 3 Department of Psychology, Alma Mater Studiorum—University of Bologna, 40126 Bologna, Italy

**Keywords:** non-technical skills, social network, safety, teamwork

## Abstract

Safety at work should be considered as the result of the daily interaction of operators. The present research wants to analyze which factors are involved in the development of social networks about safety at work. We assumed that two relational non-technical skills, such as safety communication and safety team member support, affect the in-degree and out-degree bonds of workers in social networks. One hundred and eight workers of a warehouse were the participants of the research, in which they were asked to fill out a self-reported questionnaire. Multiple linear regression analysis was used to test the hypotheses. Results confirmed that safety communication and safety support skills play a role in determining the quantity and the quality of social bonds that workers can create at the workplace. To be specific, while safety communication was found to be associated with out-degree centrality (b = 0.24; *p* = 0.01), a nonsignificant relationship was found for in-degree centrality. In contrast, safety team member support was found to be associated with in-degree centrality (b = 0.28; *p* = 0.04). In other words, on the one hand, it was found that high levels of safety communication skills are associated with the tendency of workers to proactively search for colleagues with whom they can share information about safety. On the other hand, workers with high levels of safety support skills tend to be considered as reference points in terms of safety by colleagues, who are more prone to look for their help. Implications for both scientists and practitioners are discussed.

## 1. Introduction

The statistical office of the European Union (Eurostat) revealed that the warehouse sector is particularly exposed to safety risks at the workplace, being one of the most threatening for workers’ health. In particular, in Italy, (where this study was conducted), the number of fatal accidents at work was above average compared to that recorded in other European states [1]. Data from the National Institute for the Assurance against Workplace Injuries (INAIL) confirmed the national trend, recording an overall number of fatal accidents exceeding a thousand workers in 2019 [2].

Given the threatening situation for workers’ health, workplace safety needs to be improved by analyzing, under different perspectives, the dynamics that lead to accidents and injuries. Accordingly, greater attention should be paid to non-technical skills (NTS), as their crucial role within the field of industrial safety has been demonstrated [3]. To be specific, as clearly pointed out by Beus and colleagues [4], safety skills represent a crucial factor that directly influences safety behaviors, which, in turn, are directly associated with accident and injuries rates. In other words, improving NTS is of the utmost importance to improve the overall safety levels at work [5].

Nevertheless, nowadays, safety training does not usually include training on NTS, rather it focuses on only the technical aspects of safety [6]. For example, workers in the warehouse sector are informed about the correct way to lift weights or how to drive pallet trucks safely, but they are not taught about working in teams to improve safety or manage fatigue and stress. Furthermore, while an increasing number of studies was carried out in recent years regarding the theme of NTS [7,8,9], a deep knowledge about the dynamics that lead to better safety performances is still missing.

Accordingly, this study’s main goal is to shed some light on the role of NTS in the working context, with a particular focus on safety communication and safety support, by analyzing their spread across workers belonging to the warehouse sector through social network analysis (SNA). SNA represents a useful instrument to analyze social networks at work to understand how workers socially interact. Recently, a variety of scientific evidence about the role of social networks with regard to safety has emerged, showing their relevance for a deep understanding of safety dynamics at work [10]. To our best knowledge, however, no study has analyzed the connection between NTS and social networks at the workplace. Accordingly, extensive research was carried out in scientific databases (PyschInfo, Scopus, PubMed, and Scholar), and no support for a connection between NTS and social networks was found.

Analyzing social networks at work may help to understand and to contextualize the spread of NTS among workers as a function of the social bonds that connects them. To be specific, it is important to understand if and how NTS are acquired and shared in the working context on the basis of the social interactions that characterize daily activities at work.

Accordingly, this study aims to expand knowledge about the way NTS are spread across workers on the basis of their social interactions. Findings of this study may be helpful for safety management in two main ways. First, understanding how social bonds are connected with workers’ skills would help management to target safety training by involving those workers who lack the required NTS, helping them to build strong social bonds at work and to work more safely. Second, understanding how NTS are shared among workers would help to identify key groups of influential workers (those with higher levels of skills and a greater number of social ties), favoring the spread of NTS in the entire social network.

## 2. The Role of Non-Technical Skills

Non-technical skills (NTS) can be defined as “cognitive, social and personal resource skills that complement technical skills and contribute to safe and efficient task performance” [5]. In other words, they represent the sum of soft skills that, in addition to hard skills, help workers to work safely. Examples of personal resources (NTS) involve the recognition and the management of fatigue and stress conditions or the ability to creatively find a solution to safety issues or problems.

In contrast to technical skills, NTS are less domain-specific: they can be found in almost every kind of job or working environment. They sustain safety performance in multiple and differentiated ways [11]. Therefore, NTS have been studied and identified across different working sectors (e.g., aviation, healthcare) [11,12].

This study focuses, in particular, on the following NTS: safety communication and teamwork (with a specific focus on team member support). The aim is to understand how they are spread among workers as a function of social interactions in the warehouse sector.

### 2.1. Safety Communication

Safety communication mainly refers to the exchange of relevant safety information among workers. Overall, safety communication skills include sending information clearly and concisely, including context variables, receiving information, and identifying and re-directing communication barriers [5].

The fundamental role of safety communication was underlined in several studies, which showed its impact on different safety outcomes, such as team safety performance [13] and safety climate [14].

Recently, a variety of studies analyzed safety communication through SNA. For example, Alsamadani [15] found that safety communication patterns in teams are directly connected with accident rates. Safety communication can also be influenced by workers gender, as shown by Allison and Kaminsky [10]. Through their study, the authors found that safety communication patterns tend to be more homogeneous in all-male crews than in mixed-gender crews. Finally, Albert and Hallowell [16] found that safety communications patterns are directly connected with the ability of teams to recognize hazards at the workplace. In particular, they found that the more well connected the crews (network density), the more they are able to recognize hazards collectively.

In summary, while recent studies analyzed the way safety information is shared in teams at work and related safety outcomes, no evidence about specific safety communication skills of workers and their spread in teams is available. Accordingly, understanding how safety information is shared in groups on the basis of individuals’ and teams’ safety communication skills may help to better understand how safety social networks work and how these NTS are spread in working groups.

### 2.2. Safety Teamwork

According to Flin and colleagues [5], teamwork is a multidimensional construct comprising the ability to support others, resolve conflicts, exchange information, and coordinate working activities. A recent review highlighted that safety could not be achieved in organizations without efficient and effective cooperation of workers. According to the framework proposed by the authors, safety teamwork enhances safety performances of both individuals and teams through increased levels of safety participation and compliance (for a review, see [17]).

Given the relevance of teamwork for safety outcomes, the ownership of safety teamwork skills appears equally essential. This study focuses on a specific dimension of teamwork, namely team member support, which represents a core aspect of effective cooperation among workers. According to social exchange theories [18], supporting and being supported by others induces workers to repay the support they receive, resulting in greater social ties at work and better working outcomes. In regard to this, a recent study provided evidence that behaviors of support among team members are directly associated with increased levels of workers’ satisfaction and commitment [19].

In the literature, different studies analyzed teamwork as a function of social interactions and bonds. For example, on the one hand, SNA was implemented to understand how to build the most efficient working teams [20]. On the other hand, Nunes and Ambreu [21] used SNA to determine critical success factors in working projects and found that teamwork efficiency plays a fundamental role.

While SNA was generally applied to analyze how social networks affect teamwork, this study focuses on the role of supporting skills of workers as a mean to understand how these are spread in teams and how they influence safety outcomes.

## 3. Social Network Analysis

The roots of social network theory are interdisciplinary, involving research in sociology, social psychology, and anthropology [22]. Social network analysis represents a useful quantitative method for analyzing the way workers socially interact, and it mainly comprises two elements: nodes and ties. The firsts are represented by workers within the network, while ties symbolize social connections between them. The main goal of SNA is to describe these social interactions by graphs and maps, which allows an understanding of the overall structure of working groups. Furthermore, quantitative analysis can be conducted to understand the degree of centrality of individuals in the network. In regard to this, two kinds of centrality measures can be used: inbound and outbound centrality. While the first represents the number of inbound links of a given node, the latter represents the opposite, that is, the number of outbound ties [23].

Several studies applied SNA to understand how social networks are connected with workplace safety. For example, in the construction sector, SNA was implemented to investigate the role of social network structures and dynamics concerning safety climate [24], safety communication [15], or the causes of accidents [25]. Furthermore, SNA was analyzed in the military sector to evaluate surgical team communication [26].

To our best knowledge, no study analyzed how NTS are spread among workers within social networks. To shed some light on the connections between NTS and social networks, this study applies the job performance theory [27], according to which individual performance is the result of individual-related factors, such as motivation and skills. Accordingly, social bonds at work can be seen as the result of the ability of the worker to connect with others (skill), as well as his motivation to create them. We mainly focus on skill, and we assume that safety communication and safety support represent useful skills that allow workers to create social bonds with co-workers. In particular, we assume that greater skills are associated with better bonds. In other words, we expect that workers that are more inclined to communicate about safety and that support co-workers in safety tasks will be more likely to create strong safety social bonds at work. These assumptions are also in line with the work of Flinn [5], who found that high levels of skills (teamworking in particular) produce as outcome an increase in interdependency among working groups and foster social relationships between workers.

**Figure 1:**
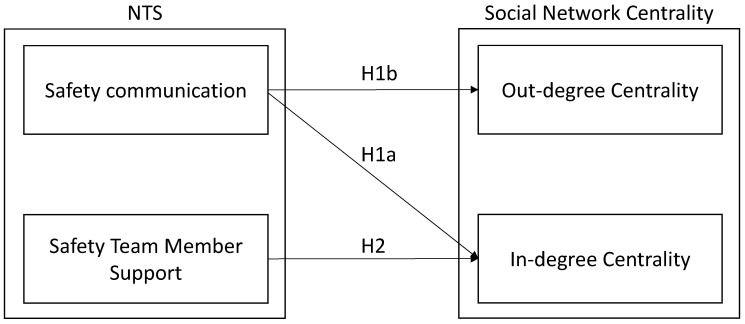
Theoretical Framework. NTS, non-technical skills.

However, we also expect a few differences in the kind of connections that these skills have with social networks. To be specific, on the one hand, we expect safety communication skills to be associated with both in-degree and out-degree ties since they are directly connected with safety information flow in the working context. In other words, we assume that workers with high levels of communication skills will be more prone to both give and receive information about safety in the workplace, favoring the development of strong social bonds with colleagues. On the other hand, safety support skills may not be directly connected with the tendency of workers to proactively exchange safety information with others, while we presume that this kind of skill is associated with the number of colleagues asking for help. In other words, we expect workers with high levels of safety support skills to represent reference points for co-workers in terms of safety. This assumption is in line with social exchange theory [18], according to which the ability to support others is directly connected with the tendency to be, in turn, supported by others through, for example, the share of relevant safety information. Accordingly, Figure 1 presents the theoretical framework of this study.

Our hypotheses are as follows:

**Hypothesis** **(H1).** *Safety communication positively predicts in-degree (a) and out-degree centrality (b).*

**Hypothesis** **(H2).** *Safety support positively predicts in-degree centrality*.

## 4. Materials and Methods

The study was carried out in a north Italy warehouse of a large-size engineering company. The warehouse was selected because it is part of a multinational company that has many warehouses, organized in three continents. Moreover, it was chosen for its organizational complexity and structure. Regarding the first, the company produces a variety of agricultural machines. The warehouse contains pieces of very different sizes and shapes: from small fasteners to wheels for agricultural tractors. In regard to organizational structure, the company is structured in two basic sectors: in-bounding (materials that are received) and out-bounding (materials that are sent outside). The working location is organized into goods receiving, merchandise storage, picking, packaging, loading, internal quality, and maintenance. In the last three years, there were six lost-time accidents and zero severe and fatal injuries.

The participants were 108 workers (73% of the warehouse population); 68% of them were male, 18% had a role in safety practices (e.g., emergency team).

### 4.1. Procedure and Measures

A structured questionnaire was used to collect the data. A trained research assistant psychologist administered the pencil-and-paper questionnaire that included statements on NTS and on the participant’s own social network for the safety. The personal data was treated in accordance with the Italian privacy law (Law Decree DL-196/2003) and General Data Protection Regulation (GDPR, UE n. 2016/679).

The Communication scale on NTS of Mariani and colleagues [3] was adopted for assessing the same construct. Since the scale was developed in chemical companies, the relevance of the items, with respect to the workplace, was checked on the basis of three interviews with safety manager and workers. There were four statements, based on a five-point Likert: (1) Communicate effectively with the supervisor; (2) Communicate effectively with colleagues; (3) Give information/feedback on your work; (4) Ask for information/feedback on your work. Following Mariani and colleagues [3], the items were introduced by the statement “Think of good health and safety practices at your workplace; how much do you feel able to contribute?”. An excellent value for the Cronbach’s alpha was registered (0.82) for the safety communication scale.

We constructed a four-item scale to measure the team member support. To do so, we analyzed the literature on the construct (i.e., [5]) and interviewed six experts (safety managers with experience in a warehouse) and three workers with an important tenure in warehouses. A list of seven items resulted from the interviews and analysis of the literature. We asked a panel of four judges (three research group members and one practitioner who had worked for more than 10 years as safety manager) to evaluate the content validity of these items by responding to the question, “Is the goal measured by this item’ essential,’ ‘useful, but not essential,’ or ‘not necessary’ to the performance of the construct?”. Consequently, four items were selected following the guidelines of Lynn [28]: (1) keep attention on your workgroup; (2) support other colleagues in carrying out work in general; (3) help resolve conflicts in the workgroup; (4) help others with homework. The four items, based on a five-point Likert, were introduced by the same statement adopted for Communication scale on NTS [3]. Explorative factor analysis, with principal axis factoring, on the data of the present research, showed that the four-item pool loaded on one factor, explaining 54.11% of the variance. A second explorative factor analysis, with principal axis factoring, on all items of NTS, showed two separate factors, one for the items of team member support and one for the items of communication construct. Furthermore, an excellent value for the Cronbach’s alpha was registered (0.90) for the safety team member support scale.

For studying the social network for the safety of participants, a question, introduced by some statements, was adopted: “In relations between colleagues, in everyday life, it sometimes happens to exchange ideas, opinions, views or suggestions on topics relating to job security. These relationships are often very useful for developing ideas and suggestions for improvement. In your experience, are there any colleagues with whom you have/more often exchange ideas, opinions, and suggestions on issues relating to job safety? Can you indicate any?” A directed network model was adopted because the relationships have an origin and a destination rather than simple mutual connections. From a methodological point of view, this represents a way to track an individual’s perceptions and mnemonic recall of social interactions. This means that the number and the quality of the mentioned interaction may suffer from cognitive and social biases.

### 4.2. Data Analysis

SPSS version 10 for Windows and Gephi were used to analyze data. The statistical analysis plan consisted of the following steps: (1) compute the index of social network analysis; (2) calculation of the descriptive statistics, and alpha of Cronbach for the analyses of the psychometrics proprieties; (3) test of the hypothesis.

Since the directed network was studied, there are two measures of degree: in-degree and out-degree. In-degree is the number of connections that point inward at a vertex. Out-degree is the number of connections that originate at a vertex and point outward to other vertices. The computed indexes of the social network were as follows: degree, as the total number of inbound and outbound connection of a single node, in-degree, as the number of connections that point inward at a vertex, and out-degree, as the number of connections that originate at a vertex and point outward to other vertices [29].

## 5. Results

### 5.1. Preliminary Analysis

**Figure 2:**
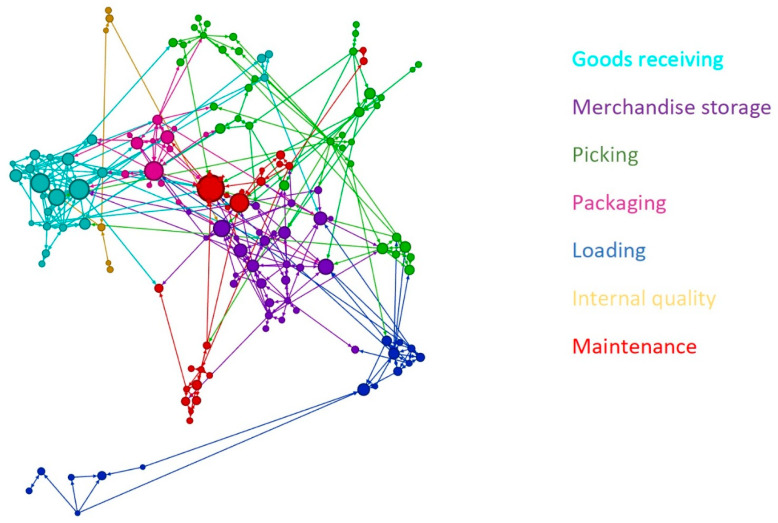
Safety communication socio-diagram.

The socio-diagram of the working network is presented in Figure 2, which is a visual representation of social interaction regarding safety in the workplace. Alongside a total of 149 nodes, 374 edges (which represent the connections between workers) were registered. The number of nodes was greater than the number of workers who fulfilled the questionnaire since they were asked to indicate the colleagues with whom they usually exchange relevant safety information: consequently, the number of nodes represents those workers who were identified by co-workers, regardless of their participation in the questionnaire. That means that, on average, workers indicated more than one colleague as a confidant about safety themes. Furthermore, it is important to point out that nodes’ size is associated with the number of in-degree bonds: the greater the latter, the bigger the node. Accordingly, it can be noticed a relevant variability in the size of nodes: this means that information exchange flow was at the same time centered on few workers (who have a greater number of in-degree bonds) and equally distributed among the majority of them (because of the smaller number of in-degree connections).

Another measure that can be used to contextualize SNA is graph density, which can be defined as the number of connections of a node divided by the maximum number of possible connections. Graph density scores vary from 0 to 1 and represent the density of information exchange processes in the network. The results showed a graph density of 2%, indicating that information exchanges were spread across the entire network.

**Figure 3:**
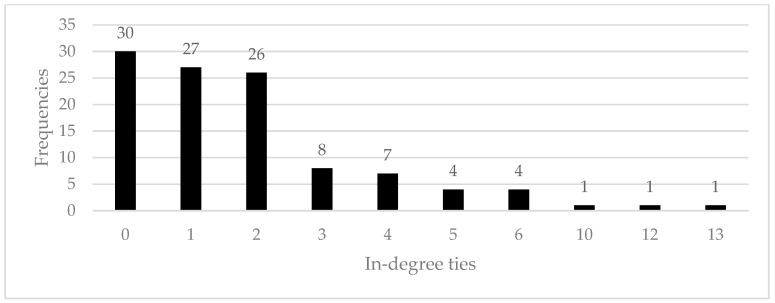
In-degree ties frequency distribution.

**Figure 4:**
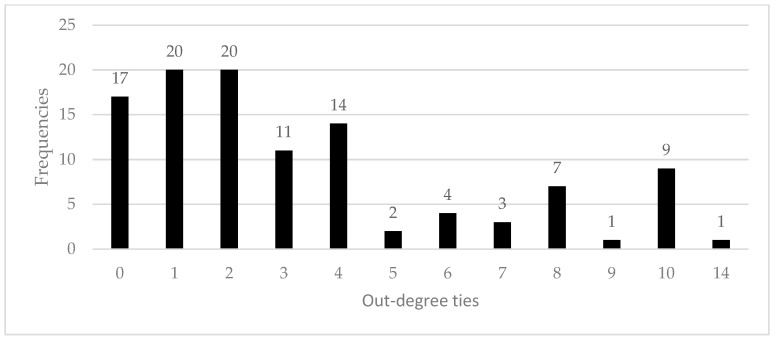
Out-degree ties frequency distribution.

Figure 3 and Figure 4 show frequencies of in-degree and out-degree bonds in the social network sample. According to the results, most workers (76.1%) acted as a reference point for safety matters to a maximum of two colleagues, while a minority (23.9%) were usually referred to by three or more of them. Concerning out-degree bonds, similar considerations can be made, since most of the workers (75.2%) usually referred to a maximum of four other colleagues for safety issues, while a minority referred to five or more co-workers (24.8%). In other words, results show that workers were usually referred to a few other colleagues for safety matters, indicating that the information-exchange processes were usually conveyed in small groups.

Moreover, it is important to underline that every worker had at least one tie with a colleague: it means that there were no cases of social isolation in the sampling social network.

The means, standard deviations, and correlations were computed for all study variables, as reported in Table 1. Relationships between variables were in the expected direction. All reliability measures of scales (Cronbach’s alpha) were above α = 0.80 and thus met the criterion requirements [30].

Overall, positive correlations between variables were found. Safety communication was positively related to both safety support (r = 0.70; *p* < 0.01) and out-degree centrality (r = 0.24; *p* < 0.05).

Safety support was also found to be related with in-degree centrality (r = 0.20; *p* < 0.05) and out-degree centrality (r = 0.20; *p* < 0.05). Furthermore, a positive correlation was registered between in-degree and out-degree centrality (r = 0.27, *p* < 0.01).

### 5.2. Model Testing

Results showed that safety communication positively predicted out-degree centrality (*b* = 0.24; *p* = 0.01). Furthermore, concerning in-degree centrality, results indicated that the dimension was positively predicted by safety support (*b* = 0.28; *p* = 0.04), while nonsignificant results were found for safety communication. In other words, results showed that communication and support skills of workers were directly connected with their safety social ties at work. However, these skills had different influential effects on the social bonds of workers. To be specific, safety communication positively predicted the number of outbound ties, whereas safety support was associated with the inbound ones.

Results of regression analysis are presented in Table 2 and Table 3. So, all the hypotheses of this study were confirmed.

## 6. Discussion

The aim of this study was to analyze social networks at work in terms of safety and their relationship with the non-technical skills of workers. Results confirmed that safety communication and safety support skills play a role in determining the quantity and the quality of social bonds that workers can create at the workplace. To be specific, on the one hand, it was found that high levels of safety communication skills are associated with the tendency of workers to proactively search for colleagues with whom they can share information about safety. This skill allows workers to strengthen their bonds with co-workers, increasing the number of out-degree connections between them. On the other hand, safety support plays a crucial role as well. Results showed that the dimension is directly associated with the number of in-degree ties. In other words, workers with high levels of safety support skills tend more to be considered as reference points in terms of safety by colleagues, who are more prone to look for their help.

Overall, the hypothesis was confirmed, although not completely. To be specific, we expected to find a connection between safety communication and in-degree centrality. However, this assumption was not confirmed by the results, since they show that only safety support is associated with the number of in-bound ties. To sum up, results indicated that while safety communication is an important NTS with regard to the number of out-bound ties, it does not seem to be a relevant factor with regard to the number of in-bound ties, which seem to be influenced by other factors such as team member support.

Nevertheless, results of this study are in line with Campbell’s theory [27], and its application in the safety field (i.e., [31]), according to which individual performance (i.e., the number of ties a worker can create and maintain at work) is the product of the individual’s ability (i.e., NTS). In other words, the ability to create social bonds in terms of safety at work is predicted by individual NTS. Furthermore, it must be acknowledged that different NTS have a differentiated impact on social networks. For example, we may expect safety leadership skills to be associated with in-bound and out-bound ties (since leaders tend to represent landmarks for co-workers), while we may assume safety awareness to be associated only with the number of in-degree bonds (since this skill might be considered as a requested ability in social networks and thus induce workers to refer to those colleagues with high levels of safety awareness).

Finally, in regard to the construct and content of the two NTS: the correlation index between safety communication and team member support is high, but its R-squared shows a proportion of the common variance of less than 50%. All that is in line with the theoretical suggestions of Flinn and colleagues [5], who presented the NTS as associated with each other, and with the results of Mariani and colleagues [3] that showed high correlation indexes among NTS dimensions.

Moreover, in support of considering the two NTS dimensions as distinct, the results underline that the two NTS predict different outcomes of the SNA, showing different roles in safety networking. In our opinion, this supports the decision to divide them into two distinct categories.

### 6.1. Theoretical and Practical Implications

Results of the study have both theoretical and practical implications. First, from a theoretical point of view, this study expands our knowledge about the way safety networks are created and developed at work. Recalling Campbell’s theory [27], it was demonstrated that workers’ ability to build and strengthen safety social bonds at work relies on their NTS, such as their ability to exchange information about safety with colleagues and to support others in safety tasks.

In line with social exchange theory [18], this study revealed that being able to support others is directly associated with an increased number of social bonds at work and, in particular, with out-degree ties. This result implies that social exchange theory can be used to link workers’ skills with the structure of social networks at work. Therefore, it is recommended to further investigate the relationship between these with the aim of understanding how different typologies of skill influence the overall structure of social networks.

From a practical point of view, this study revealed that SNA could be a useful tool to understand how social boundaries about safety are built at work. This kind of information can be used by organizations as a mean to strengthen the social bonds of workers and consequentially increase the overall levels of safety at work. Furthermore, once NTS levels in the network are detected, organizations can use this information to identify those workers who both have higher skills and play central roles in the safety network to further expand NTS skills among them. For example, identified workers might be asked to share with co-workers their knowledge and skills about safety or they might be involved in dissemination projects aimed at increasing NTS in safety social networks.

From the workers’ viewpoint, in addition to an improvement of safety performance, the acquisition of NTS can be a useful means by which to strengthen social bonds at work. For instance, improving safety support skills may help workers to be more involved in social networks about safety and, as a consequence, favor a constant development and update of safety skills and performance.

Increasing the overall levels of NT2 might be beneficial for both workers and organizations [32], because of their impact on several safety outcomes such as, for example, safety performance [6], safety participation [33], and safety climate and culture [34]. This study allows an understanding of how NTS are spread across social network and thus allows a focus on the connections between workers as a means by which to improve the overall levels of safety at work. In other words, granting an equal possession of NTS among workers would translate into better safety performances and with a lower number of accidents and injuries.

### 6.2. Limitations and Future Research

This study presents some limitations. First, results were collected through a cross-sectional design, which represents a limitation since it does not allow precise causal relationships among the variables to be determined. Future research should focus on overcoming this limitation through longitudinal designs, which will allow a better understanding on whether and how safety social networks are influenced by workers’ NTS.

Second, alongside the cross-sectional design, this study only focused on a single organization of the warehouse sector, thus figuring as a case study. This represents a limit for multiple reasons. First, safety dynamics characterizing a specific working context may differ among organizations. Second, this study was carried out in an organization operating in the warehouse sector; thus, results may not be directly extended to different working contexts. Future research should try to overcome these limits by analyzing the relationship between NTS and social networks in different sectors and organizations. Furthermore, while the company is dislocated on an international level, this study was conducted in an Italian plant, implying a poor generalizability of results to other countries or other plants of the company itself. Future research should try to overcome this limit by analyzing the spread of NTS in different organizational and cultural contexts.

Another limit about the specific context of this study relates to the social network structure of the company. It must be pointed out that the structure of a social network is highly influenced by workers characteristics and behaviors. For example, psychological research suggests that workers tend to create social bonds with colleagues that are somehow similar to them [35]. This phenomenon—known as homophily—may contribute to the development of clustered social networks, in which workers relate only with similar others, thus hindering the spread of information across the network [36]. Since this study did not analyze the potential influence of communication obstacles or barriers in social networks, we highly suggest future research to pay attention to groups’ characteristics in order to analyze their impact on the spread of NTS among workers.

Third, SNA was implemented as a means by which to understand connections between workers’ roles in social networks and their NTS levels. However, we focused only on two specific parameters, in-degree centrality and out-degree centrality, as reference points for our analysis. Future research should try to analyze how NTS relate to different social networks indicators, such as network density or betweenness and closeness. Furthermore, while we studied the role of safety communication and team-member support in social networks, other NTS may be relevant as well, such as safety leadership or safety awareness.

Fourth, NTS were analyzed through self-assessment measures. These represent a limitation since individual perception about one’s own ability may differ from others’ evaluations. Therefore, future research should try to analyze differences between self-assessment and co-workers’ assessment of NTS.

## 7. Conclusions

This study shed light on the connections between NTS and safety social networks in a warehouse. To be specific, it was demonstrated that NTS levels play a fundamental role in determining the quantity and quality of safety bonds that workers create and maintain at work. Moreover, findings of this study suggest that different NTS typologies produce a diversified impact on social networks: while safety communication is directly related with the number of outgoing social connections, safety support relates to the number of incoming ties. Results thus indicate that safety social networks at work are shaped around workers’ skills, which are heterogeneously spread on the basis of their social interactions. These findings contribute to understanding how to contextualize interventions aimed at improving NTS among workers by providing decision-makers with useful insights about the best way to share knowledge and skill in social networks. At the same time, this study provides scientists with increased theoretical knowledge about social dynamics related to the acquisition and spread of NTS in working networks. Accordingly, we suggest to further analyze the structure of social networks with the aim of understanding how individua and group characteristics influence its development in the working context.

Starting from these results, future research should try to expand knowledge about the dynamics of safety in working contexts by further analyzing the role that NTS play in social networks and their practical and theoretical implications.

## Figures and Tables

**Table 1:** Means, standard deviation, and correlations among study variables.

Variable	M	SD	R		
			1	2	3
1. Safety communication	3.94	1.0	(0.82)		
2. Safety support	3.68	1.0	0.70 **	(0.90)	
3. In-degree centrality	0.01	0.02	0.09	0.20 *	-
4. Out-degree centrality	0.02	0.02	0.24 *	0.20 *	0.27 **

Note: * *p* < 0.05; ** *p* < 0.01; M = mean; SD = standard deviation; in brackets = Cronbach’s alpha.

**Table 2:** Relationship between safety communication and support with in-degree centrality: regression analysis.

Predictor	Unstandardized Coefficient		Standardized Coefficient	*t*	*Sig.*
	Β	SE	β		
Constant	0.004	0.006		0.71	0.48
Safety communication	−0.002	0.002	−0.11	−0.86	0.40
Safety support	0.004	0.002	0.28	2.05	0.04

Note: SE = standard error; R^2^ = 0.005; R^2^ adjusted = 0.002.

**Table 3:** Relationship between safety communication with out-degree centrality: regression analysis.

Predictor	Unstandardized Coefficient		Standardized Coefficient	*t*	*Sig.*
	Β	SE	β		
Constant	0.002	0.008			
Safety communication	0.005	0.002	0.24	2.58	0.01

Note: SE = standard error; R^2^ = 0.06; R^2^ adjusted = 0.03.

## Data Availability

Not Applicable.
